# Children’s perceptions about falls and their prevention: a qualitative study from a rural setting in Bangladesh

**DOI:** 10.1186/1471-2458-13-1022

**Published:** 2013-10-29

**Authors:** Salim Mahmud Chowdhury, Leif Svanström, Lars-Gunnar Hörte, Rabiul Awal Chowdhury, Fazlur Rahman

**Affiliations:** 1 Division of Social Medicine, Department of Public Health Sciences, Karolinska Institutet, 171 76 Stockholm, Sweden; 2 Centre for Injury Prevention and Research, Bangladesh (CIPRB), Mohakhali, Dhaka 1207, Bangladesh

**Keywords:** Perception, Falls, Children, Prevention, Bangladesh, Rural

## Abstract

**Background:**

Childhood falls is a major public health problem in Bangladesh. In-depth understanding of the situation by the target groups and their families is necessary for successful development, implementation and evaluation of any intervention. The study aimed at knowing the views of Bangladeshi rural children about childhood falls and their suggestions for prevention.

**Methods:**

Children of 10–17 were selected purposely from 4 villages of Sherpur Sadar upazila (sub-district), Sherpur district of Bangladesh. Six focus group discussions and ten in-depth interviews were conducted during July-August 2010 for this study. Gender and education of the participants were considered. Major themes were identified, coded and categorized from content analysis.

**Results:**

Participants stated that young children (<5 years of age) and boys appeared to be the main victims of falls and majority of these injuries occurred in and around the households. Boys commonly fall from the tree around their premises and high places. Girls usually fall when they remain busy in household chores and playing with friends around their premises. Participants also mentioned that children mostly sustained injury when they are unsupervised. Supervision, public awareness and putting barriers (e.g. door barrier, putting pillow and use net around the bed etc.) were suggested as the preventive measures.

**Conclusion:**

Findings of this study could be considered as part of knowledge-base in designing interventions to address childhood falls.

## Background

Childhood falls and their complications is a leading cause of the global burden of disease [1]. While falls don’t rank in the top 15 causes of death for children under 15 years, they rank 13th as a leading cause of disability-adjusted life years (DALYs) [2]. Globally, non-fatal falls account for between 25-52% of all medically treated injuries among children [3,4]. A recent epidemiological study from Bangladesh established falls as the 4th leading cause of morbidity after infancy and rural children are at great risk [5]. Study on medical help seeking behaviour in Bangladesh has shown falls from trees was the main cause of hospitalisation of children due to trauma [6].

The magnitude of falls like other unintentional injuries varies according to the age, sex, region, income group, cultural background, and kind of injury [7,8]. Children’s psychological characteristics influence injury occurrence as injuries are the result of specific interactions between the behaviour of children and the surrounding environment [9,10]. Investigation of the context and mechanism are an essential part of the childhood falls prevention knowledge base like other injuries [11]. In recent years, it has been observed that injury knowledge base was complemented by the qualitative research [12] along with quantitative information. In-depth understanding of situation by the target groups and their families is necessary for the successful development, implementation and evaluation of programmes [13]. Although, there is a study on parents’ perception about child injury [14] but there is no study on child’s attitude, believes and behaviour toward falls in context of rural Bangladesh. Even, to the best of my knowledge there is no such study at global level. This study was conducted as part of an initiative to develop a comprehensive falls prevention programme for the rural Bangladeshi children. Hence, the study aimed to gain in-depth understanding of the event by exploring perception of falls among Bangladeshi rural children and their suggestions for prevention.

## Methods

This study is a qualitative investigation of the rural Bangladeshi children’s perceptions about falls and their risk taking behaviour along with their prevention practices. Participants for this study were selected randomly considering gender variation stratified by school and non-school going children. Children who go to school and who don’t, both have the similar probability to fall. However, school going and non-school going children may have different opinions regarding occurrence and prevention of falls due to their exposure to different environments and level of knowledge. Hence, both groups of children were considered. Two different qualitative tools were used for this study - In-Depth Interview (IDI) and Focus Group Discussion (FGD). A guideline (with prompts) was developed to follow the process; relevant probing was considered based on grounded clues or questions. Both IDI and FGD were chosen for this study to cross check their opinions as what one agree/disagree in front of his/her peer, may not be his opinion while s/he asked individually especially regarding sensitive data like, sex, addiction, caring, ethical practices etc. The Ethical Review Committee of the Centre for Injury Prevention and Research, Bangladesh has given approval to carry out this study as an operational research under PRECISE (Prevention of Child Injuries through Social Intervention and Education).

### Site information

This qualitative study was conducted in 5 villages of Bajitkhila union (lowest administrative unit) of Sherpur Sadar upazila (sub-district), Sherpur district. Bajitkhila union of Sherpur Sadar upazila, Sherpur district was purposively chosen since the area can be characterised as typical representative of rural Bangladesh. Villages for this study were selected considering the size, population density and demographic heterogeneity of the inhabitants of the village after consultation with different stakeholders.

### Interview tools development and content

The interview tools were developed based on literature review and expert opinions on prevalent risk factors for childhood falls. Experts reviewed the tools before pre-testing concerning the scope and coverage of the study. Range of tools allowed for the triangulation of findings, thus enhancing the interpretation of the data and facilitating our ability to validate information as the study evolved. Extensive training was conducted for the junior research fellows (anthropologist) on the use of the final tools after necessary correction following pre-testing. Free flow of opinions and conversation was encouraged for the study.

### Participants’ characteristics

It was evident that a child’s self-reported participation in decisions concerning his/her own activities increases sharply between ages 10 and 14 years [15]. So, children of 10–17 years from the selected villages were included for this study and the total number of participants for this study was 52. Six FGD and ten IDI were included in the study: FGDs were conducted in three villages out of selected five villages. Two FGD (one boy’s and one girl’s) were conducted in each village. In-depth interviews were conducted in all selected villages (one boy and one girl in each village). Usually FGD is time and cost effective method and more participants view comes through FGD but its arrangement is little bit difficult. Population and geographic characteristics were homogenous among all selected villages. Thus, we conducted FGDs in three villages. Table 1 shows the different tools used for this study along with participants’ characteristics.

**Table 1:** Tools and characteristics of participants employed for data collection

**Tools applied**	**Sex/characteristics of participants**	**No. of FGD/IDI**
Focus Group Discussion (FGD)	School going boys	2
	Non-school going boys	1
	School going girls	2
	Non-school going girls	1
In-depth interview (IDI)	School going boys	3
	Non-school going boys	2
	School going girls	3
	Non-school going girls	2

### Process

The focus group discussions (FGD) with the children were conducted at resident premises with 8 to 10 participants in each group. Children of a ‘Para’ (cluster of households) were considered for each FGD. FGDs with non-school going children were conducted in the morning, while FGDs with school going children were conducted in the afternoon (after school hours). In selecting participants, preference was given to those who had heard of or seen any falls related event but it was not mandatory. One trained teams consisting of one facilitator, two note-takers and one organiser were deployed to conduct the FGDs. After welcoming participants in each session during FGD, the facilitator gradually introduced a series of prompts to explore the desired information. A guideline was used for the in-depth interviews. All the questions used for both FGD and IDI were open ended i.e*.* what do you know about falls, how can it be prevented etc. Before the interview or discussion, the aims of the study were explained to the participants as well as to their parents, confidentiality guaranteed, and permission asked to record the interview, thus obtained informed verbal recorded consent.

The study was carried out during July and August 2010. Facilitators conducted the interviews and the focus group discussions in the preferred language of the participants. All interviews were recorded and verbatim transcription done. Data analysis process followed the manual qualitative data analysis. We called this ‘manual content analysis’ where the contents were first identified from the guideline and prompts, than cross checked were done with the findings and themes were identified. Finally, themes were interpreted by the core investigators where facilitators’ feedbacks were taken regular basis.

## Results

### Children’s views on falls

A very common observation about falls from the children’s statements was that “falls are a sudden mishap that can result in terrible injury”. A few participants from both the IDIs and FGDs considered that falls are the cause of many mental and physical problems.

### Socio-demographic characteristics

Overall, it was apparent from the IDIs and FGDs that children aged 0–4 years are the most vulnerable to falls. Most of the participants commented that children aged 0–4 years are very curious and want to touch and grab everything in their surroundings; hence they fall. Only a few participants reported that boys of 12–14 years are most vulnerable to falls. A common response from both IDIs and FGDs was that boys are more vulnerable than girls, though both are at risk of falls. Boys like to climb trees, play and run all the time, so they are at greater risk of falling down. One of the children (a boy) said: *“Cheleder football khela, cricket khela, gase chora, cholafera beshi, aijonno amader jhuki beshi”.* [“*Boys play football and cricket, climb trees and move frequently, so that we (boys) have a greater risk of falls*”]*.* Another said: *“Meyder jhuki kom, cheleder beshi. Chelera shob kaje beshi thake, ai jonno oder pore jaber jhuki beshi. Meyra halka patla, pore na”.* (“*Boys are at more risk than the girls. They are involved in various activities and are at more risk of falls. Girls are not in the risky group because they are skinny*”)*.*

A few participants in FGDs and IDIs, mainly girls, commented that girls are more obedient towards their parents. They usually follow their parents’ instructions, and have less risk of falling than boys. Girls, who do not go to school, remain busy with household chores and have less risk of falls. About one third of the participants, mainly girls, mentioned that children from affluent families have less risk of getting injured because of falls.

### Mechanism of falls

Most of the participants reported that children aged 0–4 years fall from the bed while trying to get down. They can fall against the door barrier if it is in their way. A few participants reported that children aged 0–4 years are curious to touch water, thus they might fall into it. Generally young children (<5 years of age) fall while they run with their friends; they can even fall while trying to push each other.

All participants reported that boys can fall from trees because of slipping, or owing to broken branches; they may also fall on the ground while playing football and cricket. Boys like to push and squeeze (hug very tightly) each other when playing, and often sustain awful injuries because of falls. A few participants reported that some boys like to sit on the roof of a moving bus. They try to push each other, which may cause massive injuries if they fall. A good number of participants from both IDIs and FGDs reported that boys and girls use vans to go to school. They often shove each other to get to the seat first; hence they fall on the ground. A small number of participants from the FGDs reported that both boys and girls can fall while trying to get off a moving bus. Boys also fall from the rooftops of houses or buildings while flying kites.

Almost all the participants from FGD and IDI reported that girls commonly slip and fall while they assist their mothers in household tasks. The majority of participants stated that girls usually play *gollachut*, *bouchi* and *kanamachi* (traditional Bangladeshi games played by mostly rural girls; these games involve mostly moving and running) and *lafdori* (another traditional game played by jumping with a piece of rope), and sometimes they sustain injuries from falls. One of the female participants said:

*“Bouichi khelar shomoy doure jaitasi, tokhon niche it baindha pore gelam, tokhon hat pa vanglam*.” (“*While playing bouchi and running fast, suddenly a piece of brick appeared under my feet, and then I fell down and my hand and leg was broken…*”).

A few participants reported that children can also fall from a table or chair while they attempt to get something from up high.

### Usual places of falls

All of the participants reported that children aged 0–4 years commonly fall from the bed and against the door. Sometimes, they go to the pond unsupervised and fall into the water. Children can also fall from chairs and tables. All participants reported that girls commonly fall on the concrete around the tube-well and yard, and that older boys commonly fall from trees around their premises. They also fall into the pond from trees, bridges and other high places. Boys frequently fall on the playground. A few participants stated that children can fall from rooftops of houses, other buildings and stairs. Anyone can slip and fall on the road and yard while stepping on fruit or food wastage.

### Time of falling

A large proportion of the participants stated that young children commonly fall when their parents are absent, mostly in the morning and at noon. A few reported that children aged 0–5 years start jumping and playing after getting up from sleeping in the afternoon, and can fall then. Most of the participants from FGD and IDI believed that girls commonly fall at noon and in the evening, while they are busy with household chores. They also fall in the afternoon while playing with friends. Nearly all of the participants said that older boys regularly fall at noon and in the afternoon, because they climb trees, dive into the pond, or play during that time.

Boys and girls going to school can slip on the road, and can even fall from the bus when they go to school, or return. They can also fall at tiffin break. Boys not attending school are at risk of falling all the time, especially when they go to and from work. Most of the participants reported that anybody can slip and fall in the dark at night, and during the rainy season.

### Persons accompanying children at the time of falls

Most of the participants noticed that boys and girls are accompanied by their friends at the time of sustaining injury caused by falls. Sometimes children are accompanied by their parents at the time when they fall, but this is not a usual occurrence. Young children (aged 0–5 years) are usually unaccompanied at the time when they fall.

### Reasons for childhood falls

Most of the participants from FGD and IDI said that parents are incapable of looking after their children all the time, because they have to go to work. Mothers cannot look after their babies all the time. A few participants from the FGD stated that sometimes they fell down, although they walk more carefully during the rainy season. While watching snakes climb trees, anybody can get scared and fall. One of the participants said that falls are the Gods’ wishes, and devils also have a role to play in falls.

### Suggestions for falls prevention from children

All of the participants in the FGDs and IDIs believed that boys and girls as well as their parents have to be conscious about the risk of falls, in order to prevent them. Participants also suggested a community-based meeting to create mass awareness about falls. Participants primarily proposed that parents can put pillows and *moshari* (nets) around the bed to protect their child from falling. They can lay the babies in *dolna* (a kind of playpen), so that they will not fall. Participants suggested putting fences around ponds and houses, as a falls prevention approach. A few FGD participants suggested that falls can be prevented by increasing supervision, and appointing another person to look after children when parents are absent.

Most of the IDI and FGD participants suggested that teachers inform children about hazardous conditions for falls, and falls-related injury. A few FGD participants suggested that the Government and NGOs could develop programmes related to falls, and use mass-media to raise awareness in the community.

## Discussion

The major determinants of childhood falls that emerged from all interviews and discussion were: lack of parental supervision, environmental conditions and demographic characteristics of children, consistent with the epidemiology around injury [16]. Relatively few studies have dealt with children’s perceptions regarding injuries. However, a study explaining self-reporting of behaviour in risky situations by 8-year-old children identified fate as the principal reason for the occurrence of minor injuries [17].

Age and gender are key variables in understanding childhood falls. Similar to other studies [5,18,19], this study revealed that younger children (0–4 years) were more vulnerable than older children. Younger children are at greatest risk of falling because of their urge to explore their surroundings: children have a natural desire to explore their environment [20], and to assess or react to the risk is not within their capacity. In contrast, many studies have shown that older children constitute a huge percentage of falls victims [21]. Because they undertake more challenging or risky actions, older children are also at greatest risk of falls [22]. Previous studies have found that boys are physically more active, play rougher and take greater risks; thus they have greater risks of falling than girls at all ages [14,23]; this is consistent with our findings. In Bangladesh, when girls grow older they usually start helping their mothers with household chores, whereas as boys grow older, their outside activities increase. This could be the main reason for such a finding. Further investigation on age-gender variations as per the different types of falls is warranted. From our study, it is evident that children from affluent families are at lower risk of being injured because of falls. A considerable body of literature indicates that low-income environments present greater risks of injury [5,24].

Places and mechanisms of injury differ between different age groups. For instance, infants are more likely to fall from bed and furniture, whereas older children usually fall from heights (for example, trees, roof-tops and balconies) and in playgrounds. In our study, it was found that younger children mostly fall from beds, chairs and furniture; this finding is not consistent with previous studies conducted in high-income countries [25]. It was also found that older boys commonly fall from trees around their premises, in high places and in playgrounds, which is consistent with other studies [5,14]. Previous studies conducted in high-income countries did not explore any difference in terms of place of injury between boys and girls. However, in our study, girls were found to fall around their premises, mostly in the yard and on the concrete around the tube-well. The variation between this finding and those in the studies in high-income settings is probably because of socio-economic and cultural differences. In this study, the home and its premises were identified as the most common places for childhood falls, as in other studies [5]. Hence, it has been recommended that home environments should be the prime foci to prevent childhood falls [26]. From the discussions and interviews, pushing, pulling and climbing were identified as the major mechanisms for falls, as in other studies conducted in Bangladesh [5,14].

The most alarming finding that emerged from this study is that children are mostly unsupervised when they fall. Young children commonly fall during their parents’ absence, mostly in the morning and at noon when their parents are busy with daily work. This finding is consistent with other studies [27]. In rural Bangladesh most families do not have enough resources to recruit someone to supervise their young children, and they have to leave their children unsupervised or under the supervision of older siblings or neighbours. From the discussions and interviews, it is also evident that boys usually fall in the afternoon when they climb trees, dive into the water or play. This finding is consistent with another study conducted in the same geographic context [14]. Children attending school mostly fall on the way to and from school. Hence, there is a need to consider children going to school in the development of falls prevention programmes.

As can be seen from the results, participants mentioned a number of approaches, including erecting barriers for the prevention of childhood falls. Most of the participants emphasised supervision, education and community awareness. Lack of parental supervision was indicated as a risk factor for many unintentional childhood injuries [28]. Most childhood injury prevention behaviour requires supervision [12]. However, there is no consistent research to support supervision or parental ability in preventing injury [29]. It was obvious from the study that supervision is a very important issue for childhood falls prevention, while at the same time different communication channels could be used for raising community awareness. Previous studies have found that a comprehensive effort is needed to address child injury by education, engineering including environmental modification, and enforcement of safety laws and procedures. In contrast, many authors have found that educational interventions have had less success than those employing passive measures [30,31]. Thus, further research is needed to develop and evaluate a feasible childhood falls prevention programme in the context of rural Bangladesh.

### Limitation

Before drawing conclusions, we must explain the study’s limitations and strengths. This is the only qualitative study to date in the context of rural Bangladesh. However, there are several limitations. First, the study was conducted in one rural area of Bangladesh, thus the results may not reflect the situation in general for Bangladesh. Finally, we could not precisely identify age-specific information on childhood falls prevention programmes following the discussions and interviews. Since types and mechanisms of injury, including injury outcome, vary according to age, further investigation in this context is recommended. Despite this limitation, the study is an important step towards a childhood falls prevention programme for rural Bangladeshi children. The open-ended process provided participant-generated rather than researcher-generated information, which is an important knowledge base for the development of appropriate interventions. Thus, we feel the information on children’s perceptions about falls reported in this study is acceptable, though there were some limitations.

## Conclusion

There is an urgent need to address childhood falls because of their huge burden at both an individual and community level in rural Bangladesh. Hence, future interventional studies should consider detailed epidemiological aspects and potential differential effects of interventions by child age, gender, and social variables in their design, analysis, and reporting. While further evidence is awaited, the existing health care system could play an important role in providing falls safety education as part of their strategies to improve child health. However, all interventions and education for childhood falls must be based on the available science, be well planned using sound public health principles, and be rigorously evaluated.

This study reveals important insights into children’s knowledge and perceptions regarding childhood falls, and their suggestions for effective prevention. Findings of this study will help injury prevention experts to further explore the determinants of childhood falls, and design interventions to address the problem in the context of rural Bangladesh, as well as for similar socio-cultural settings in other low-income countries.

### Ethics

The Ethical Review Committee of the Centre for Injury Prevention and Research, Bangladesh has given approval to carry out this study under child injury prevention project of the organization.

## Competing interests

The authors declare that they have no competing interests.

## Authors’ contributions

All the authors were directly involved in concept development. SMC, RAC and FR provided technical support in finalizing tools and methodology for this study. RAC worked directly with the data collectors for identifying, coding and categorizing major themes. SMC along with RAC drafted the manuscript. All the authors reviewed the manuscript, provided feedback to revise the manuscript and give permission to submit the manuscript for publication. All authors read and approved the final manuscript.

## Pre-publication history

The pre-publication history for this paper can be accessed here:

http://www.biomedcentral.com/1471-2458/13/1022/prepub
